# Predicting dengue transmission rates by comparing different machine learning models with vector indices and meteorological data

**DOI:** 10.1038/s41598-023-46342-2

**Published:** 2023-11-05

**Authors:** Song Quan Ong, Pradeep Isawasan, Ahmad Mohiddin Mohd Ngesom, Hanipah Shahar, As’malia Md Lasim, Gomesh Nair

**Affiliations:** 1 Entomology Laboratory, Institute for Tropical Biology and Conservation, Universiti Malaysia Sabah, Jalan UMS, 88400 Kota Kinabalu, Sabah Malaysia; 2 Faculty of Computer and Mathematical Sciences, Universiti Teknologi MARA, Perak Branch, Tapah Campus, 35400 Tapah, Malaysia; 3 Centre for Communicable Diseases Research, Institute for Public Health, National Institutes of Health, Ministry of Health, Shah Alam, Malaysia; 4 Entomology and Pest Unit, Federal Territory of Kuala Lumpur and Putrajaya Health Department, Jalan Cenderasari, 50590 Kuala Lumpur, Malaysia; 5 Phytochemistry Unit, Herbal Medicine Research Centre, Institute for Medical Research, National Health Institute, Setia Alam, Malaysia; 6 School of Electrical and Electronics Engineering, Universiti Sains Malaysia, Penang, Malaysia

**Keywords:** Computational models, Data mining, Machine learning, Infectious diseases

## Abstract

Machine learning algorithms (ML) are receiving a lot of attention in the development of predictive models for monitoring dengue transmission rates. Previous work has focused only on specific weather variables and algorithms, and there is still a need for a model that uses more variables and algorithms that have higher performance. In this study, we use vector indices and meteorological data as predictors to develop the ML models. We trained and validated seven ML algorithms, including an ensemble ML method, and compared their performance using the receiver operating characteristic (ROC) with the area under the curve (AUC), accuracy and F1 score. Our results show that an ensemble ML such as XG Boost, AdaBoost and Random Forest perform better than the logistics regression, Naïve Bayens, decision tree, and support vector machine (SVM), with XGBoost having the highest AUC, accuracy and F1 score. Analysis of the importance of the variables showed that the container index was the least important. By removing this variable, the ML models improved their performance by at least 6% in AUC and F1 score. Our result provides a framework for future studies on the use of predictive models in the development of an early warning system.

## Introduction

Dengue fever is the most widespread mosquito-borne disease, and the global incidence has increased dramatically over the last five decades^1,2^. The World Health Organisation (WHO) estimates that more than 100 million dengue incidences occur annually^3–5^, and the disease is endemic in all tropical countries^2,5^. Due to global warming and urbanisation, nearly 75% of people in the Asia–Pacific region are at risk of infection^6^, and the number of countries where dengue fever has been identified is increasing.

The disease is transmitted by the primary vector *Aedes aegypti* (L.) and the secondary vector *Aedes albopictus* (L.), which are highly adapted to urban environments^7^. In endemic countries, WHO recommends the use of vector indices such as the house index (HI), Breteau index (BI), container index (CI) and premise index (PI) as a quantitative tool to estimate vector abundance to improve dengue surveillance. According to WHO^8^, to calculate these indices, the container index is the percentage of containers containing larvae or pupae. The premises index (PI) is the percentage of positive premises with larvae or pupae out of the number of premises inspected. The house index is the percentage of houses infested with larvae or pupae. The Breteau index (BI) is the number of positive containers per 1000 inspected houses. Previous studies have found an association between the indices and dengue transmission. For example, Sanchez et al.^9^ assessed the discriminatory power of BI and HI as predictors of dengue outbreak and found a strong association between indices and transmission with the highest area under the curve (AUC) of 0.71. Chadee^10^ used retrospective data from Trinidad and showed that PI and CI were significantly associated with dengue incidences. Morales-Perez et al.^11^ assessed HI, BI, CI and PI at household and cluster levels of dengue transmission and found that BI was the only weak predictor of dengue infections. The results of these studies show that the indices can potentially be used as variables to predict dengue transmission rates. Indeed, both vector abundance and dengue transmission have seasonal patterns that are strongly influenced by meteorological factors^12,13^. Therefore, meteorological data have often been used to develop a model to predict dengue transmission rates. For example, Anwar et al.^14^ used an empirical model with ambient temperature to predict dengue incidence in some high-risk countries. Masrani et al.^15^ used five weather data—minimum temperature, average temperature, maximum temperature, rainfall, and wind speed—as predictors in a generalised additive model (GAM) to predict dengue incidence in northeast Malaysia. Xu et al.^16^ investigated the direct and indirect effects of temperature and precipitation on dengue outbreaks in Guangzhou China using a structural equation model (SEM).In previous studies, other algorithms were used to develop the predictive model. For example, the Bayesian Markov Chain Monte Carlo (MCMC) technique was used to build a model to predict the dengue outbreak in Singapore and Honduras^17^. Supervised machine learning (ML) algorithms have also been explored to address the challenge. For example, Sarma et al.^18^ used machine learning algorithms, namely decision trees (DT) and random forest (RF) and achieved an average accuracy of 79%. Salim et al.^19^ developed Support Vector Machine (SVM), DT and Artificial Neural Networks (ANN) to predict dengue transmission outbreak in Selangor Malaysia using climate data and achieved the highest accuracy of 70% using SVM algorithm. Roster et al.^20^ found that DT ensemble models with epidemiological and meteorological variables gave more robust results in predicting monthly dengue cases in Brazil. Nevertheless, most studies have not adequately considered such appropriate disease variables in modelling^21^, focusing mainly on meteorological data^11–20^, or used only vector indices to develop a logistic regression model^22^. Moreover, other ML algorithms that was demonstrated robust in prediction, such as XG Boost, AdaBoost and Random Forest have not been investigated for their ability to predict dengue transmission. Therefore, in this study, we aim to develop a dengue transmission rate prediction model that uses both vector indices and meteorological data, and compared seven machine learning algorithms were most commonly used in predictive studies^23^ for their discriminative power in dengue outbreak classification.

## Materials and method

### Data set construction

To create the dataset, we obtained the vector indices from the Entomology and Pest Unit, Health Department of Federal Territory of Kuala Lumpur & Putrajaya, Malaysia. These include house index (HI), Breneu index (BI), container index (CI) and premise index (PI) from January 2018 to December 2020 in five districts, namely Titiwangsa, Kepong, Cheras, Lembah Pantai and Putrajaya. The meteorological data for each area, such as rainfall, maximum temperature, humidity and barometric pressure, were obtained from the Malaysian Meteorological Department. For the temperature variable, we used the maximum temperature rather than all minimum, average, and maximum temperatures together to avoid the problem of collinearity. Furthermore, the maximum temperature was able to provide a larger trend variation^24^. The target variable is dengue transmission rate, i.e., whether an outbreak occurs or not. An outbreak is defined as the transmission of dengue fever in an area in a given period with a higher frequency than expected. According to the World Health Organisation (WHO), the operational definition of a dengue fever outbreak in Malaysia is the reporting of more than two standard deviations of the 4-week average of dengue cases above the three-week moving average of dengue cases^25^. Before building the model, the data set was checked for typographical errors, outliers and missing values. Normality of the data was analysed using the Shapiro–Wilk test. A variable with a p-value of less than 0.05 for the Shapiro–Wilk test was considered not normally distributed.

### Variable importance analysis

**Figure 1:**
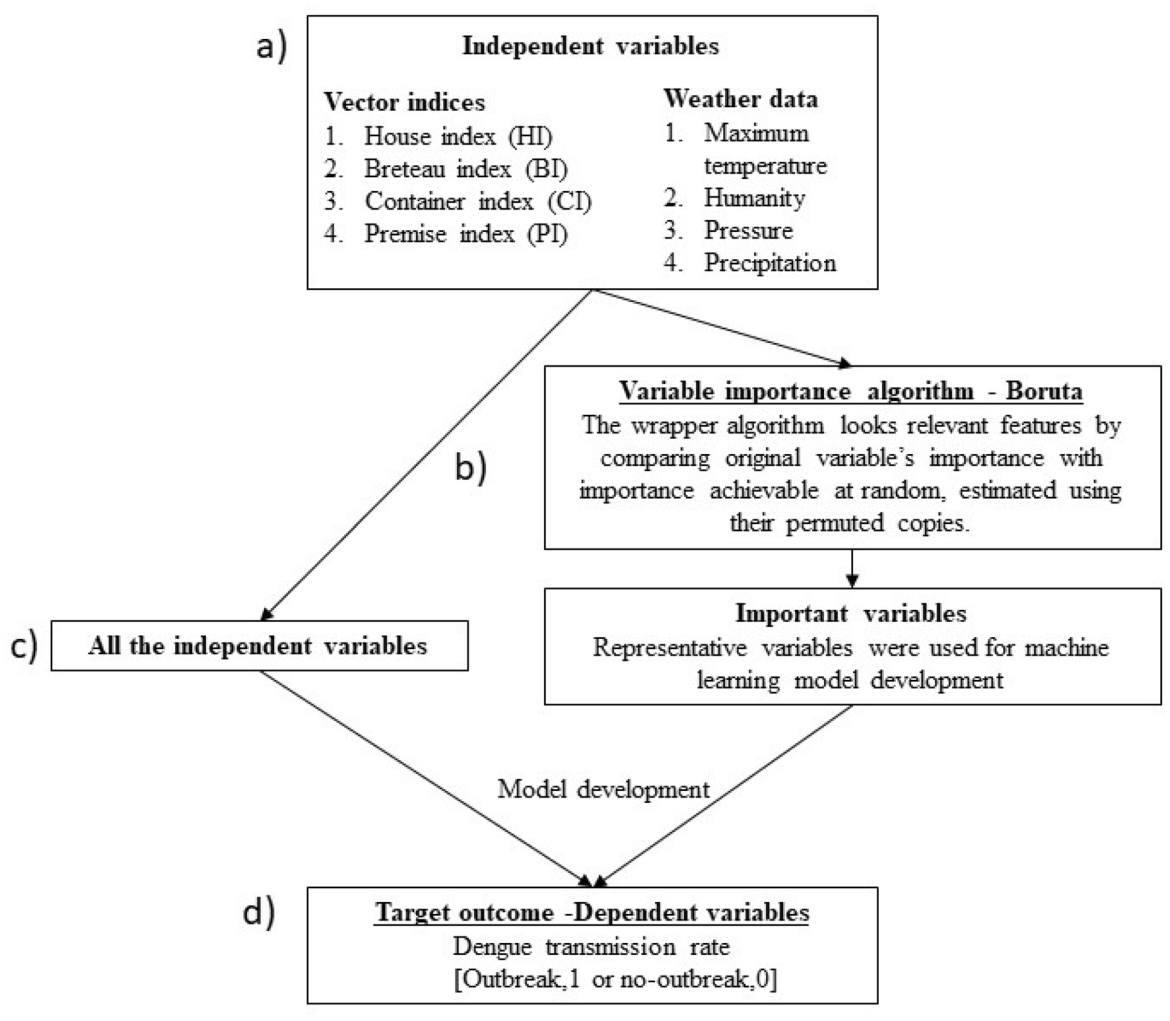
Illustration of identification of important variables using the Boruta algorithm. (**a**) All independent variables studied in modelling; (**b**) Variable selection using the Boruta algorithm; (**c**) Development of a model using all independent variables or important variables selected using the Boruta algorithm; (**d**) Prediction of the target outcome—dengue transmission rate.

We applied variable importance analysis, which is commonly used prior to modelling high-dimensional data in the development of machine learning models^26,27^. The process of selecting study variables with the greatest predictive power for dengue transmission rates. Variable selection improves model performance by eliminating redundant features, reducing computational costs, and reducing overfitting when analysing high-dimensional data^27^. In this study, we use the Boruta algorithm^28^, a wrapper algorithm to select all relevant features. Figure 1 illustrates the process of variable importance analysis using the Boruta algorithm. The random forest-based algorithm finds relevant variables by comparing the meaning of the original variables with the randomly achievable meaning estimated from their permuted copies^27^.

### Model development

**Figure 2:**
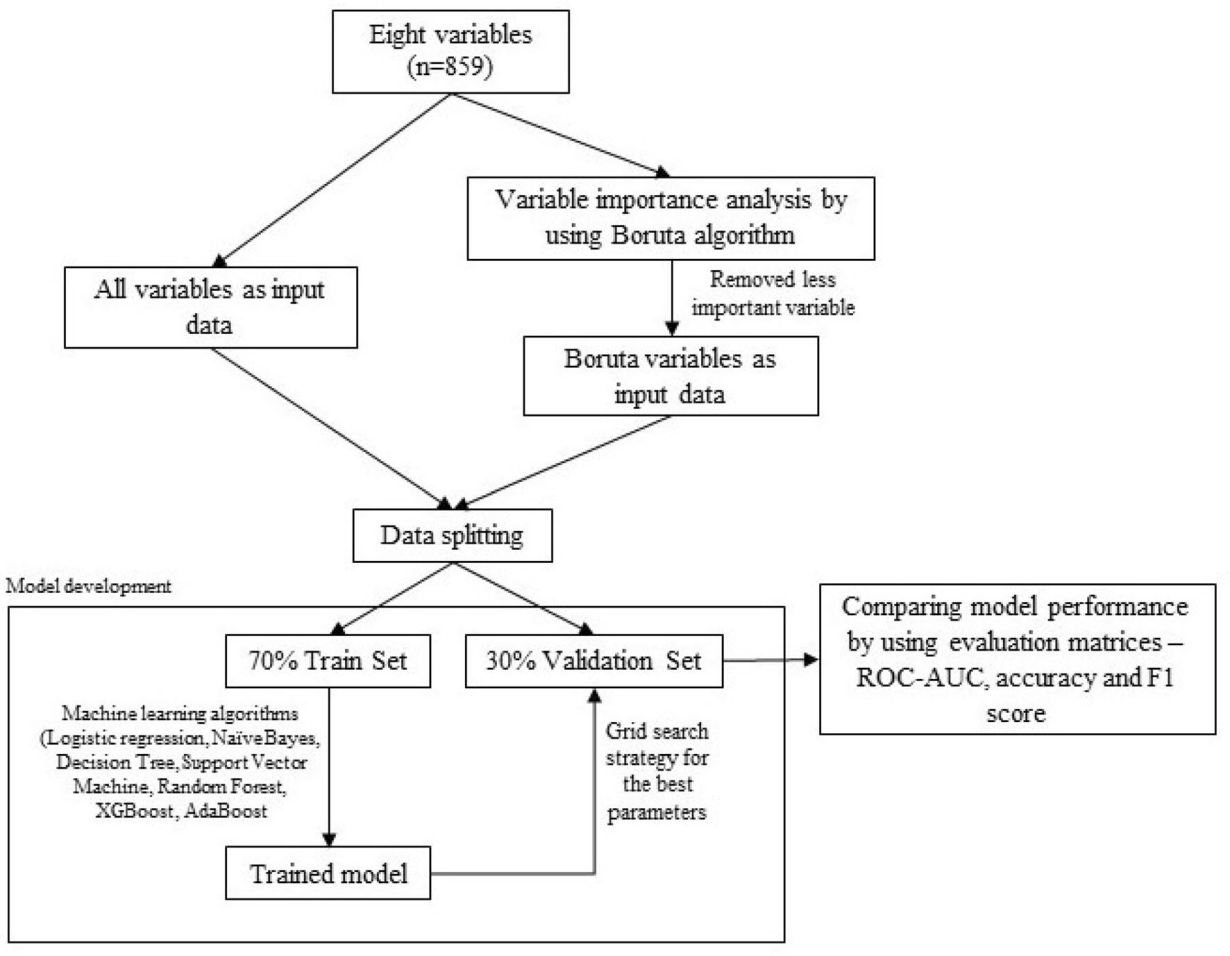
Workflow for the development of machine learning classifiers using all variables or Boruta variables.

Figure 2 shows the workflow of developing a machine learning model (ML) for dengue transmission rate classification using two sets of input data, namely all variables and Boruta variables, respectively. We built several predictive models using ML algorithms (logistic regression [LR], decision tree [DT], random forest [RF], support vector machine (SVM), XGBoost [eXtreme Gradient Boosting] and AdaBoost [Adaptive Boosting]). We split the study data into 70% (*n* = 602) for model training and 30% (*n* = 258) as a validation set in which the trained model was used to predict this unseen data.

To evaluate the performance of the model, we plot the Receiver Operating Characteristic (ROC) curve, which represents the sensitivity to specificity of a classification model and obtain the area under the curve-AUC (Table 1)^29^. We also used standard evaluation matrices for precision (1) and the F-measure, also known as F1-score (2), which can be simply interpreted as a harmonic mean of precision and recall (Table 1) and is often used to evaluate the performance of classification algorithms^30,31^.Grid search strategy^32^ was used to optimise the model to find the best hyperparameters and parameters to predict the target output of the model. Grid search strategies exhaustively generate candidates from a grid of parameter values specified with the algorithm parameters (e.g., *C*-value for logistic regression and minimum leaf samples for decision tree). Later, the candidates that fit the data set are evaluated with all possible combinations of parameter values and the best combination is selected.

**Table 1:** Formulas for calculating the evaluation matrices from a confusion matrix.

Evaluation metrics	Calculation or Equation
AUC	
Accuracy	$\frac{{{\text{TP}} + {\text{TN}}}}{{{\text{TP}} + {\text{TN}} + {\text{FP}} + {\text{FN}}}}$(1)
F1-score	$\frac{{2 \times \;{\text{precision}}\; \times \;{\text{recall}}}}{{{\text{precision}}\; + \;{\text{recall}}}}$(2)

*True Positive (TP); False Positive (FP); False Negative (FN); True Negative (TN) and precision is calculated by [TP/(TP + FP)] and recall is calculated by [TP/(TP + FN)].

### Ethical statement

This study has been registered with National Medical Research Register with ID RSCH ID-22-04904-PMV for using the vector indices that collected by Department of Vector Control, Ministry of Health Malaysia.

## Result

### Dataset

**Figure 3:**
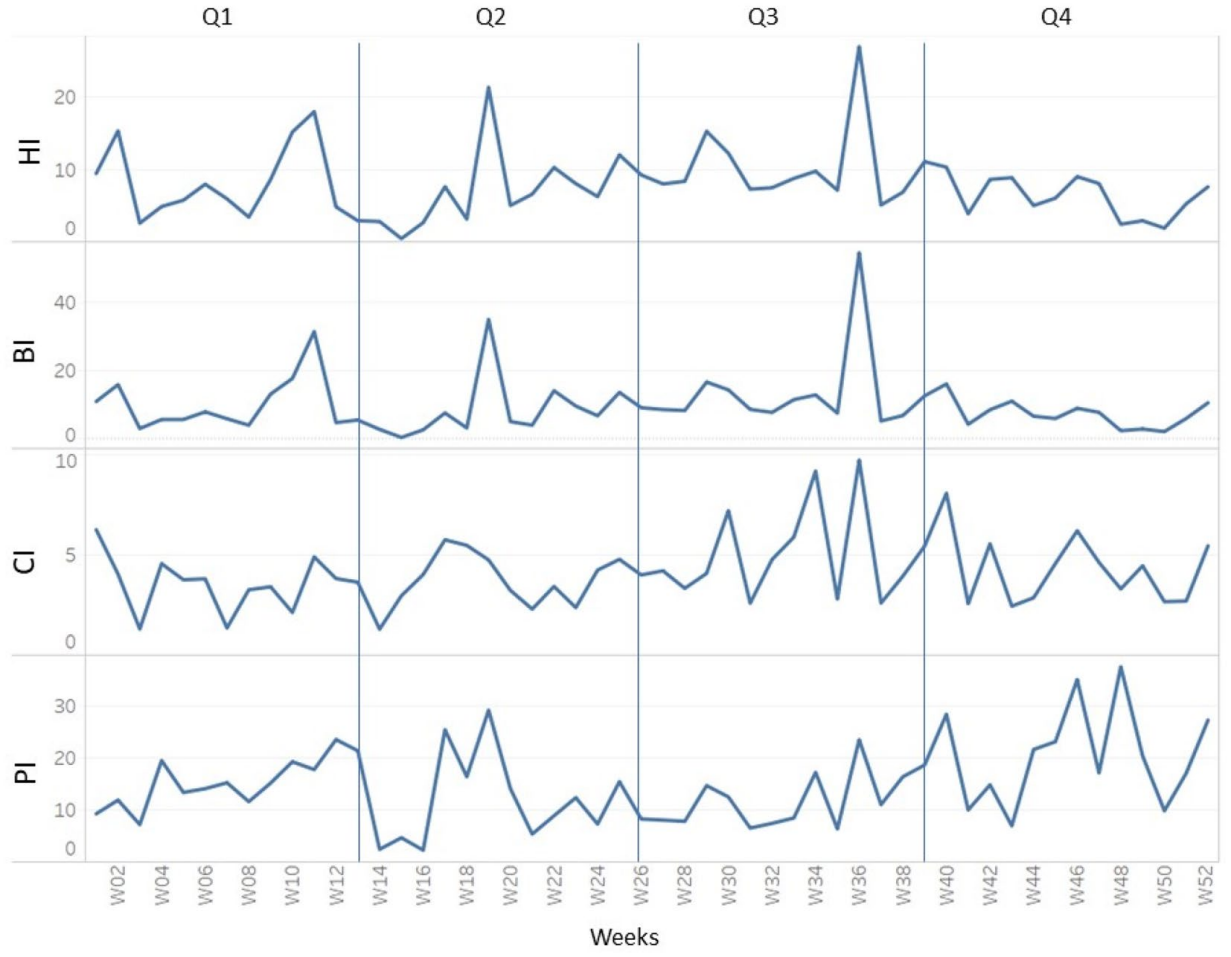
Temporal trend of vector indices.

**Figure 4:**
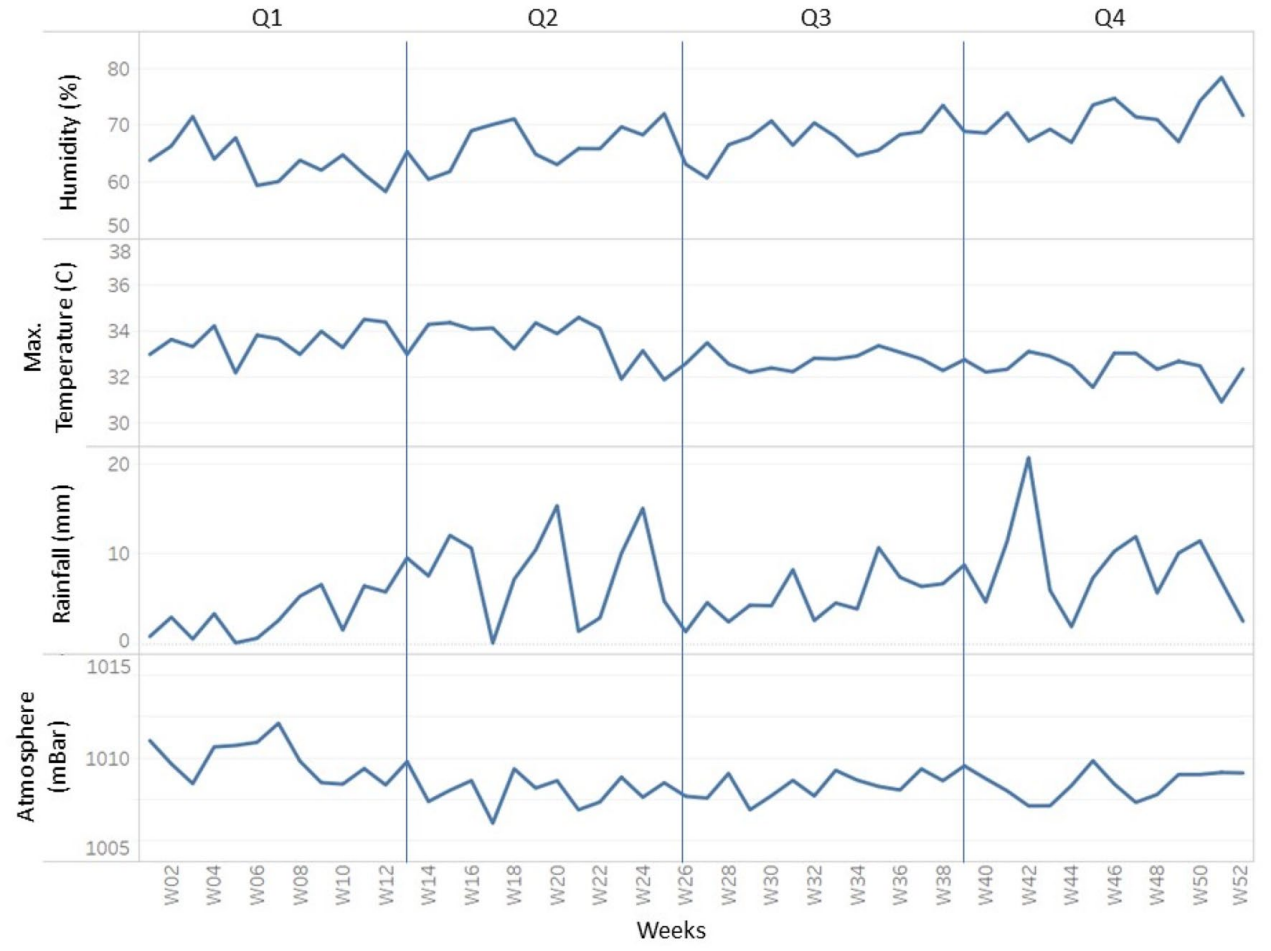
Temporal trend of meteorological data.

**Figure 5:**
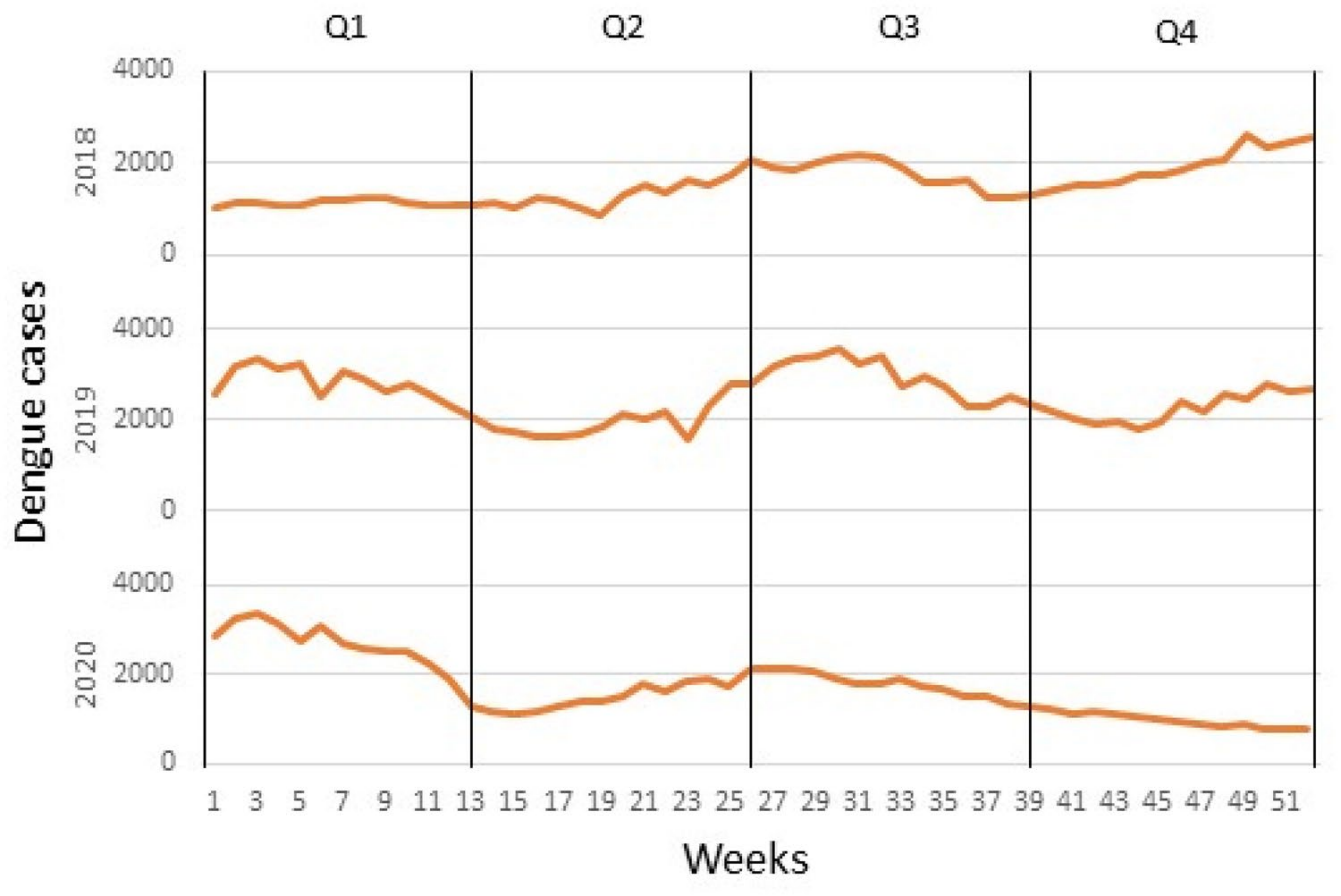
Temporal trend of dengue cases from 2018 to 2020 by quartile.

In this study, a dataset with a total of eight variables was created, containing vector indices and meteorological data from five districts of Federal of Kuala Lumpur, Malaysia. Table 2 describes the variables in the dataset used in this study. Figures 3 and 4 show the weekly temporal trend for the average of vector indices and meteorological data from 2018 to 2020. As shown in Fig. 3, -Q2 and Q3 generally had higher indices than Q1 and Q4, and the pattern of vector indices was more similar to the rainfall pattern (Fig. 4). It is noteworthy that the vector indices each show their own seasonal trend, even though they all measure immature *Aedes* mosquitoes. This suggests that the variables are not collinear and certainly have different predictive power. To visualise the temporal trend of dengue transmission rates, the temporal trend of dengue cases was shown in Fig. 5.

**Table 2:** Variables Analysed in this study.

Variables	Units	Description and values
House index	%	Numerical
Breteau index	–	Numerical
Container index	%	Numerical
Premise index	%	Numerical
Maximum temperature	Celsius	Numerical
Humidity	%	Numerical
Atmosphere Pressure	mBar	Numerical
Rainfall	mm	Numerical
Dengue transmission rate	Binary Boolean	0_Yes 1 No

### Variable importance analysis

**Figure 6:**
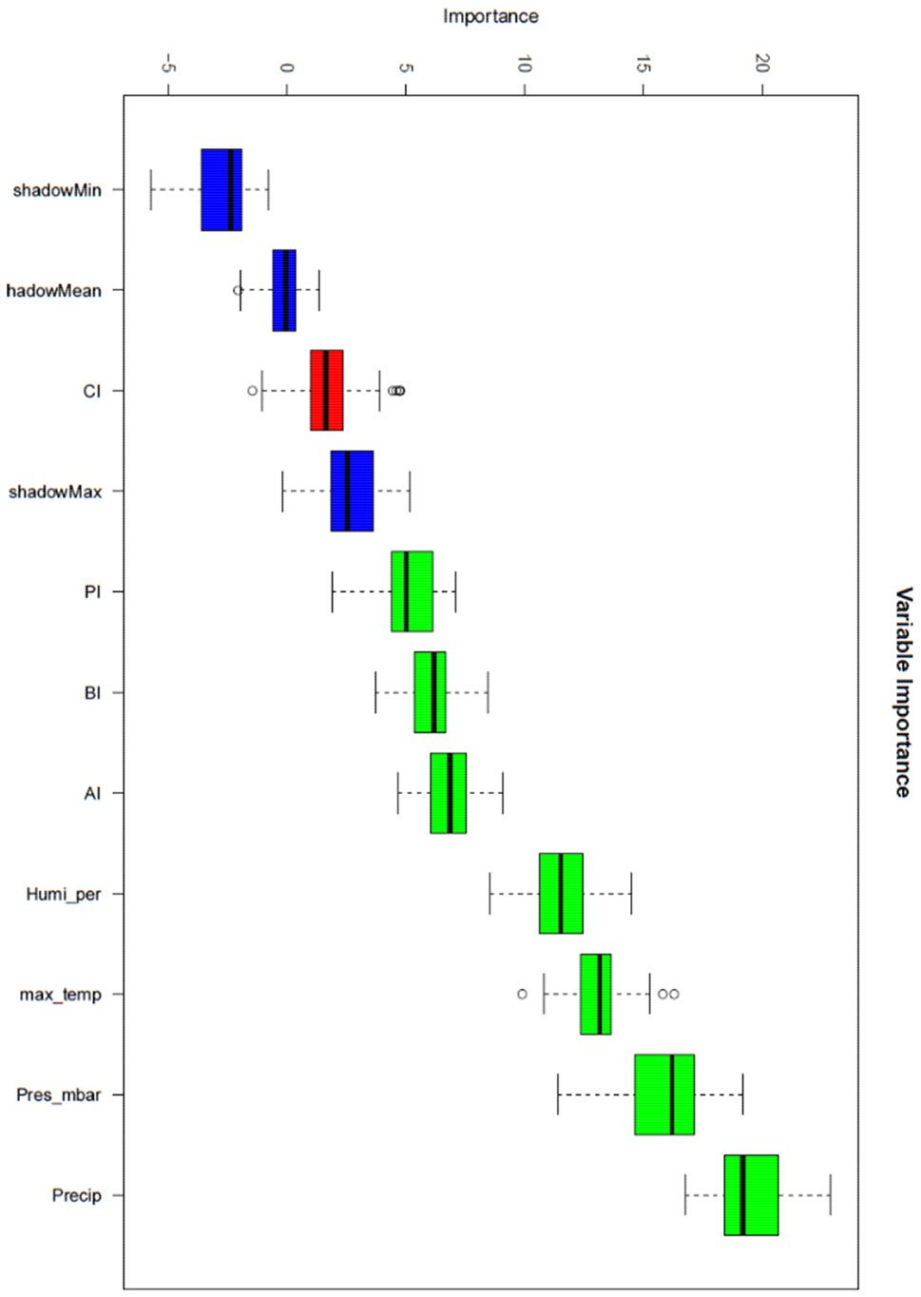
Boruta result plots for all variables. Blue boxplots correspond to the minimum, average and maximum Z-score of a shadow attribute. Red and green boxplots represent Z-scores of rejected and confirmed attributes, respectively.

In general, meteorological data were more important than vector indices; HI is the most important vector indicator. It is noteworthy that CI was listed as the less important variable (Fig. 6). Therefore, this variable was filtered out and the rest was used to develop the machine learning model.

### Performance of the model

**Figure 7:**
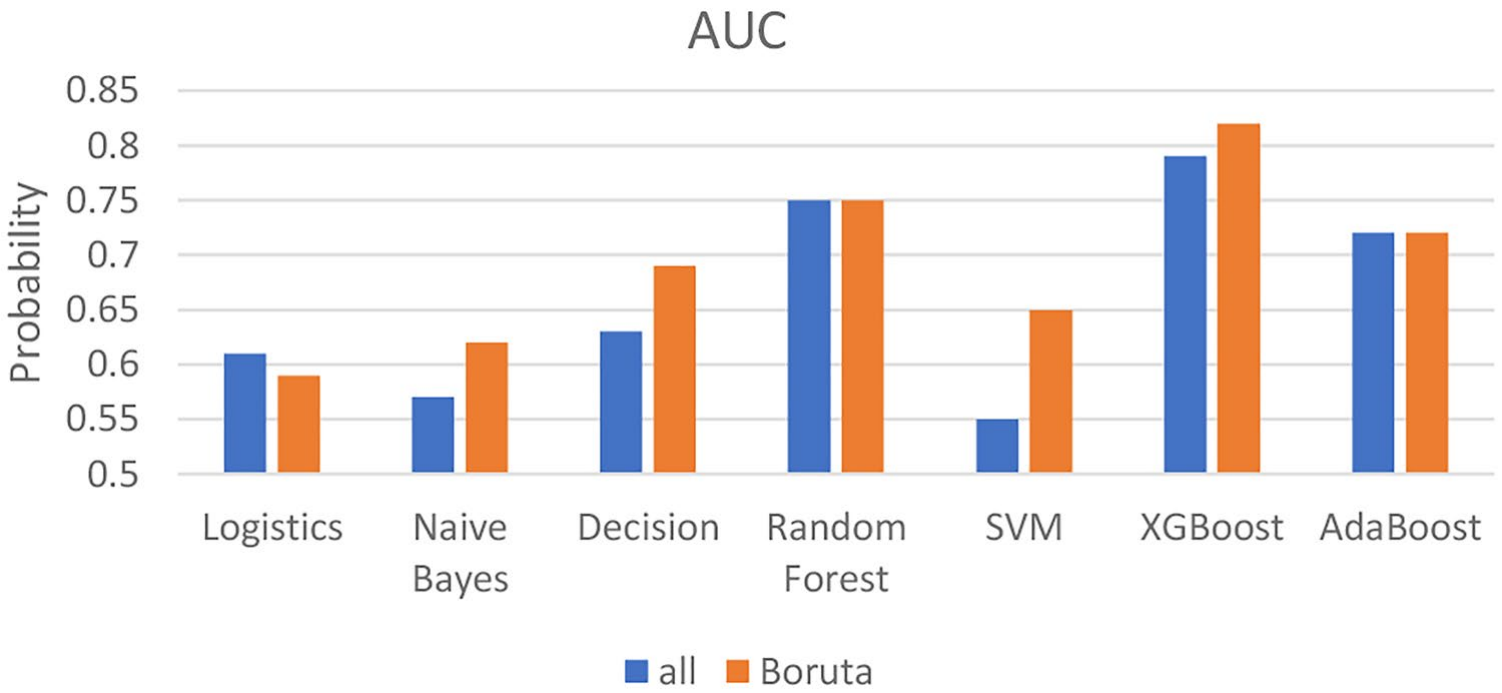
Comparison of AUC performance of the machine learning model using either all or Boruta variables.

In order to develop a predictive model to differentiate dengue transmission rates, two groups of variables, namely all variables and Boruta variables, were used as input data for model development. Using the Boruta variables, Naïve Bayes, DT, SVM and XGBoost had an average 6.71% higher AUC than using all variables (Fig. 7), indicating that the Boruta algorithm improved the performance of the model.

**Figure 8:**
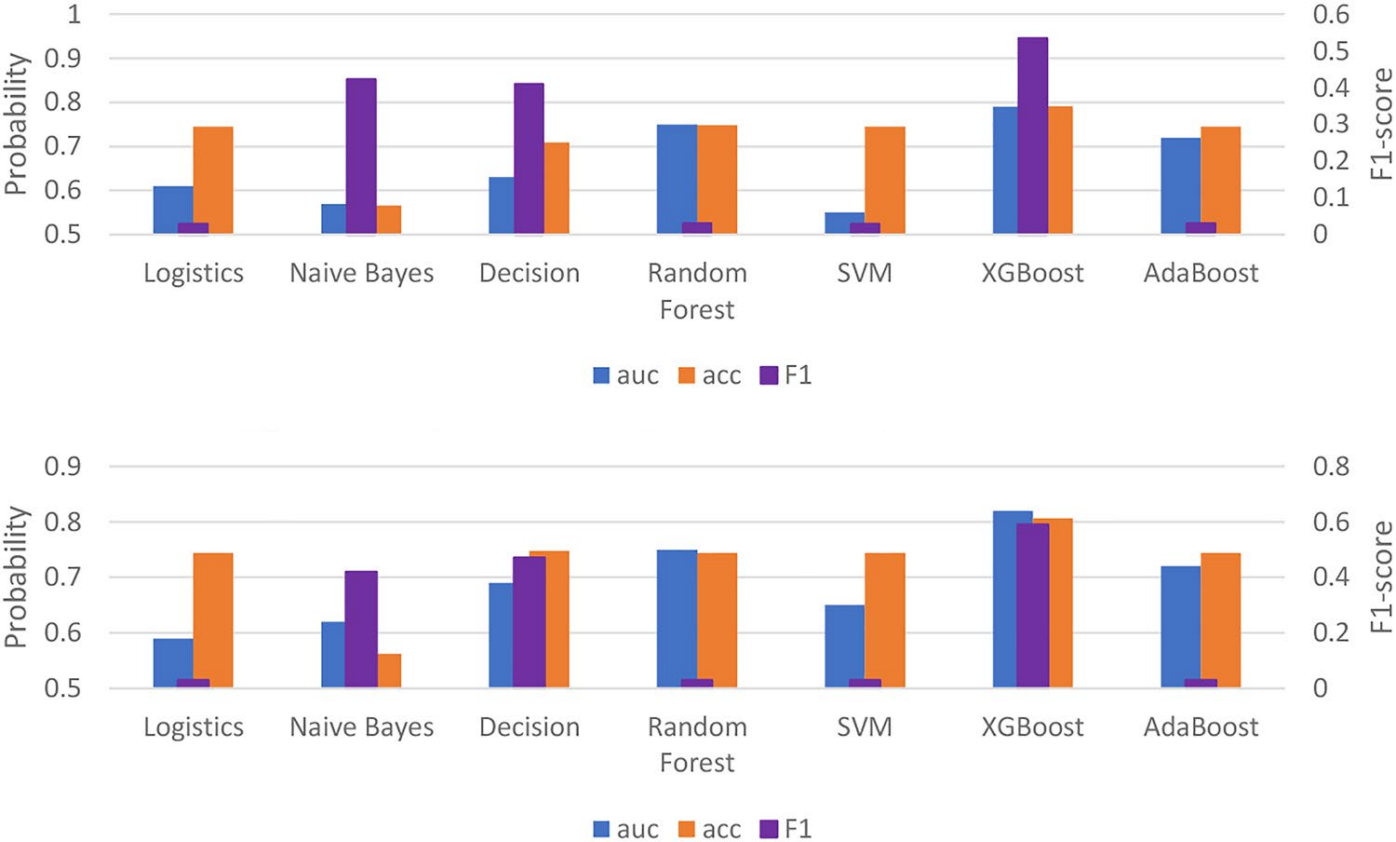
The result of the performance of machine learning models (ML) using all variables (top) and the variables selected by Boruta (bottom).

We are interested in which type of machine learning (ML) classifier is able to predict dengue transmission rate. Therefore, a total of seven ML classifiers were compared with their model performance. Figure 8 shows the result of the performance of the machine learning (ML) models using all variables or Boruta’s variables. The ensemble methods, including Random Forest, XGBoost and AdaBoost, generally performed better than the other ML algorithms. Specifically, using Boruta's selected variables, the XGBoost algorithm performed best in AUC, precision and F1 score.

Due to the trade-off between precision and recognition, the F1-score, which has a hormonal mean between two scoring matrices, is a better tool for evaluating the performance of a classifier^29,30^. As can be seen from Fig. 8, the classifier has good performance in AUC and precision but a low F1 score. Therefore, based on these matrices, XGBoost is the ML algorithm that has the greatest performance among the algorithms in this study.

Table 3 shows the ROC-AUC and the confusion matrices for the model performance as well as the parameters obtained by using the grid search strategy. As can be seen from ROC, the ensemble learning algorithm, especially XGBoost, was able to reach the threshold or trade-off between sensitivity and specificity earlier than other ML algorithms. The parameters used for all variable models may differ from those of the Boruta variable model. This was evident in the algorithms of RF and XG, which used many parameters to generalise the result.

**Table 3:**
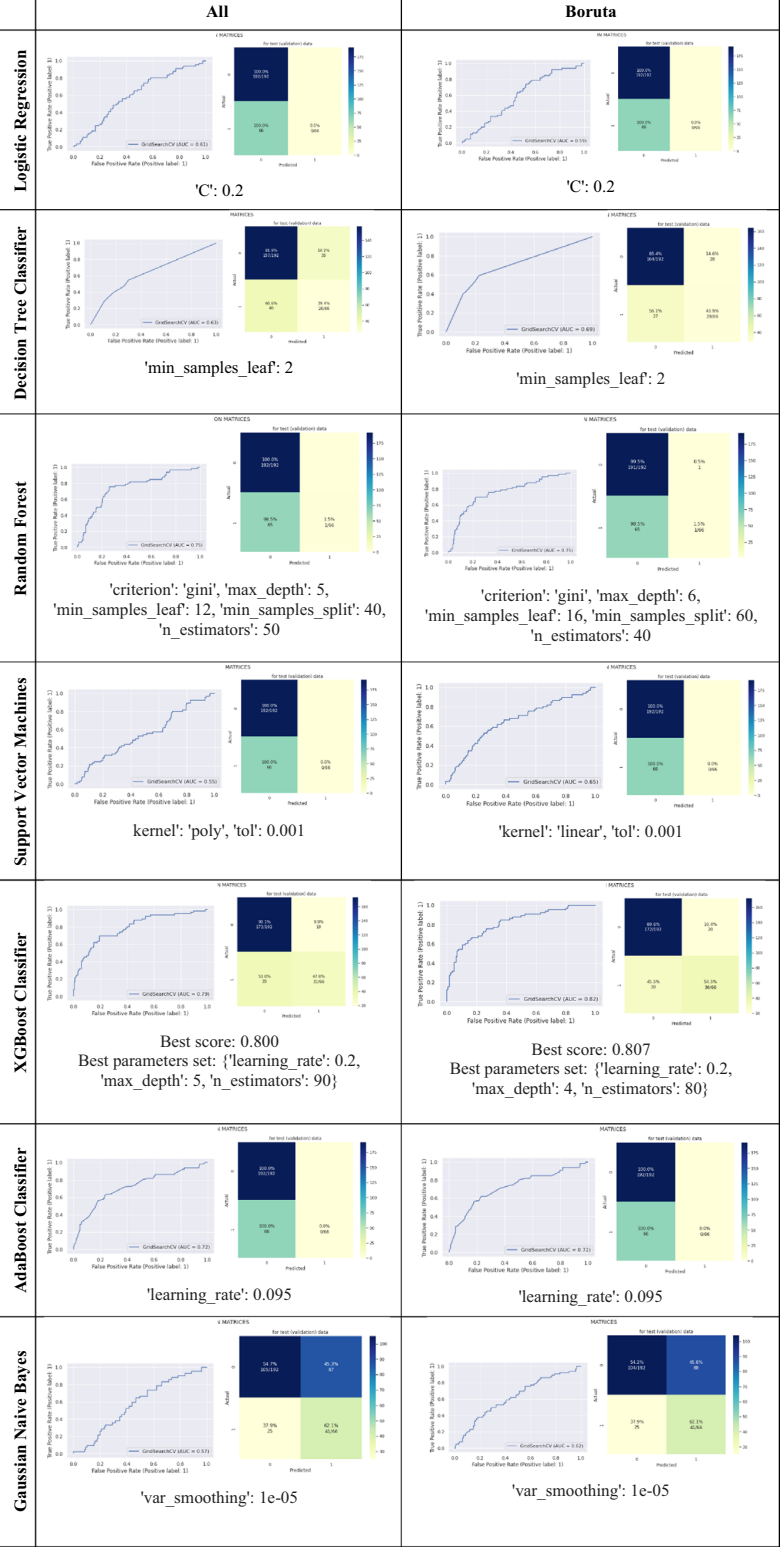
ROC-AUC and confusion matrices for model performance and parameters obtained with the grid search strategy.

TON (turnover number) = [(mmol of product formed)/(mmol of catalyst used)]

TOF (turnover frequency) = [TON/time (h)]

The TON and TOF values were calculated based on the existed amount of Zr in the nanocatalyst (in 5 mg of the nanocatalyst, 0.186 mg (or 0.00203894 mmol) of Zr has existed)

## Discussion

The magnitude of dengue transmission is increasing worldwide, making the prediction of dengue outbreaks in advance crucial. This study considered a dataset that included dengue risk factors for vectors and meteorological data, which was more complete, unlike previous studies that used either meteorological data^17–19^ or vector data^10,21^. The dataset was similar to that of Chang et al.^22^, who also used both entomological and meteorological data from 2005 to 2012 in a non-endemic city of Kaohsiung, Taiwan, but only used vector data to develop a multivariate logistic regression model.

From our result of variable importance analysis, the Boruta algorithm shows that the meteorological variables were more important than the vector indices (Fig. 6). This result is in agreement with the study of Sylvestre et al.^33^, according to which the most important predictors were precipitation, temperature and humidity. In addition, our findings that meteorological variables are stronger predictors of dengue transmission rates were also confirmed by Kamana et al.^24^, who used rainfall and temperature to predict malaria case recurrence in China. On the other hand, the vector indices we used in this study, which indicate immature populations, have been questioned by some previous studies, according to which the relationship between vector indices and dengue transmission rates is weak^2,10,34^. It is noteworthy that the container index (CI) is the only variable not listed as an important variable by the algorithm. This was also confirmed by removing the CI during the development of the machine learning model and obtaining an average improvement in AUC of 6.71%. The weak correlation between CI and dengue transmission was also mentioned by Garjito et al.^35^ who collected a total of 65,876 mosquito larvae and pupae as CI and found no correlation between the indices and dengue transmission; and Bhat et al.^36^ who conducted an entomological survey in selected villages in Tirunelveli district and found a weak correlation (R_2_ = 0.43) between CI and dengue incidences. On the other hand, the reason why the vector indices were relatively less important than the meteorological data could be that the indices refer to the immature mosquitoes that cannot transmit the virus. This reasoning is supported by many valuable studies that question the strength of vector indices, particularly Stegomyia indices, in monitoring dengue transmission. Bowman et al.^2^, for example, reviewed the literature and concluded that the majority of the literature found a weak or no association between immature vector indices and dengue transmission rates.

Nevertheless, HI, BI and PI were the important variables listed by Boruta and were used in the model development. Our result shows that vector indices can play a crucial role in improving the predictive model. This can be further substantiated by comparing our result with previous studies that used only meteorological data^17–19^ and hardly achieved more than 80% AUC. Add to these studies like that of Sanchez et al.^8^ who found that BI and HI are good predictors of dengue transmission rates with 71% AUC, 8% sensitivity and 63% specificity. We agreed that additional risk factors could be included as variables to predict dengue transmission in the future. These included adult female *Aedes* mosquitoes^2^ and host mobility. Some studies also suggested the geographical element to use a different algorithm for localisation^5^, which supports the idea of using a more flexible algorithm in the architecture of the early warning system for dengue transmission or outbreak.

Our result of predicting dengue outbreak using machine learning algorithm is encouraging. This has already been demonstrated by Salim et al.^19^ who used SVM with linear kernel to predict dengue outbreak and achieved 70% accuracy. The high performance of RF, XGBoost and AdaBoost in this study was supported by Roster et al.^20^ who studied different ML algorithms and concluded that RF could be the algorithm that helps in dengue surveillance. Our study and results also extend the ML algorithms studied by Sylvestre et al.^33^, where the decision tree and neural network performed best. However, to prevent the modelling from being more like a “black box”, we need to remember to justify and explain the mechanism within the model, e.g., the parameters used by the model, and justify the performance of the model based on the ROC and the learning curve. We should also strongly consider using more than one scoring matrix for modelling. For example, ROC-AUC is a standard and powerful matrix for evaluating the discrimination of binary classes. Accuracy is very commonly reported in modelling, but often leads to misinterpretation^37^. Therefore, we included the F1 score, which is a harmonic mean of recall and precision and balances the trade-off between recall and precision. The predictive model we have demonstrated in this study could be used in the backend of a dengue early warning system that uses meteorological data and vector indices to predict dengue outbreaks in specific areas. Or the model could be used as an algorithm in the backend of an app that allows the public to check the status of dengue transmission in their own region and possibly take some precautions and personal protection against the Aedes mosquitoes. Nevertheless, this study also has some limitations. For example, a more representative vector index such as the pupal index and the adult index might be a better indicator as it is closely linked to the adult population. However, due to the time and effort involved, this index data was not included in the dataset, which could be made up in a future study when the data is available. In summary, we have demonstrated the ability of the ML algorithm to build a predictive model for dengue transmission. Future work should be further explored in terms of feature selection, model architecture and a larger data cohort.

## Data Availability

The datasets used and/or analysed during the current study available from the corresponding author on reasonable request.
